# Congenital cytomegalovirus infection-related thrombocytopenia

**DOI:** 10.1590/0037-8682-0471-2022

**Published:** 2023-01-23

**Authors:** Melis Deniz, Mehmet Fatih Deveci, Nazlı Gülsüm Akyel

**Affiliations:** 1 Sanlıurfa Training and Research Hospital, Department of Pediatric Infectious Diseases, Sanlıurfa, Turkey.; 2 Sanlıurfa Training and Research Hospital, Department of Neonatology, Sanlıurfa, Turkey.; 3 Sanlıurfa Training and Research Hospital, Department of Pediatric Radiology, Sanlıurfa, Turkey.

**FIGURE 1:**
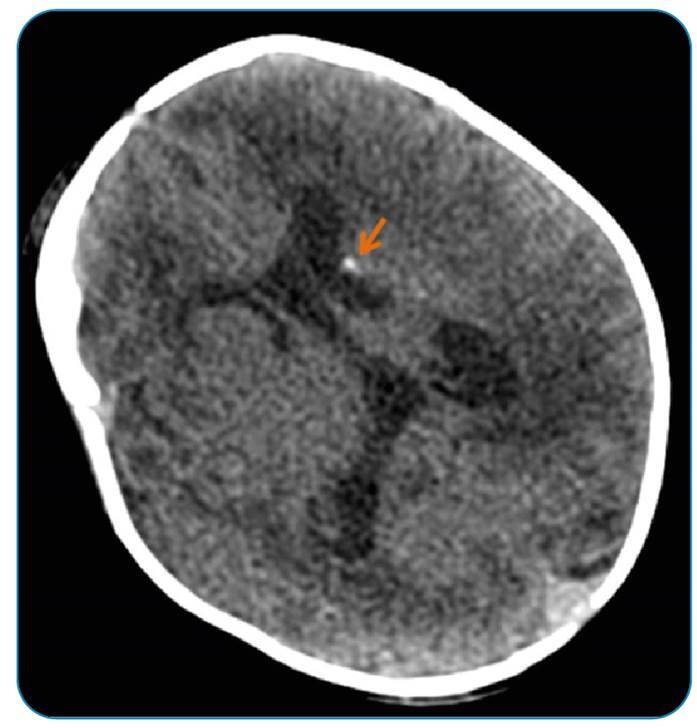
Unenhanced axial computed tomography image shows periventricular calcification (arrow) and hypodense white matter involvement.

**FIGURE 2:**
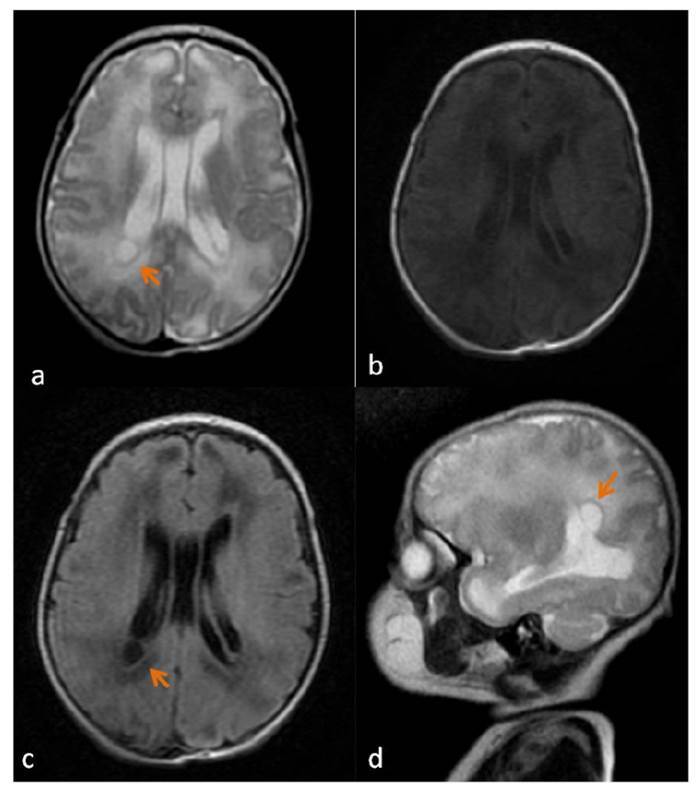
Axial T2-weighted **(a)**, T1-weighted **(b)**, FLAIR **(c)**, and sagittal T2-weighted **(d)** images show diffuse white matter signal abnormality, a finding indicative of delayed myelination. Subependymal cyst (arrows) at the right posterior periventricular region is also demonstrated.

A neonate with respiratory distress and no petechial rash was admitted to the neonatal intensive care unit. The initial blood count revealed thrombocytopenia. Results from the peripheral blood culture and additional *biochemical* tests were negative. Due to persistent thrombocytopenia, investigations were conducted for congenital cytomegalovirus (cCMV) infection. Positive results for CMV-specific immunoglobulins G and M were found on two separate occasions. Elevated blood levels of CMV DNA (4,285 copies/mL) 5 days after birth and high urinary levels of CMV DNA (1.96 million copies/mL) within 2 weeks of birth were detected. A computed tomography scan of the head detected periventricular calcifications (Figure 1). Magnetic resonance imaging of the brain confirmed bilateral periventricular white matter disease and cystic abnormalities in the right lateral ventricle (Figure 2). While the ophthalmologic evaluation was unremarkable, the hearing test revealed a moderate deficit in both ears. After initiating treatment with intravenous (IV) ganciclovir, a gradual increase in the platelet count was observed. After the platelet count stabilized, IV ganciclovir was replaced with oral valganciclovir. 

Newborns with cCMV infection may have nonspecific findings such as petechiae, decreased platelet count, hepatosplenomegaly, jaundice at birth, chorioretinitis, sensorineural hearing loss, and seizures^1^ ^,^ ^2^. Intracranial calcifications, white matter disease, cystic abnormalities, periventricular leukomalacia, and ventriculomegaly may be observed on neuroimaging^2^. Since early antiviral therapy reduces the long-term adverse outcomes (mostly hearing impairment) and neurologic sequelae, cCMV infection should be considered as one of the causes of thrombocytopenia in neonates and antiviral therapy should be started in the first month of life^3^.
